# Aging Theories and Prevention of Age-Related Diseases Using Phytocomplexes

**DOI:** 10.3390/biology15090733

**Published:** 2026-05-06

**Authors:** Marat R. Khanturin, Georgiy A. Demchenko, Laura U. Koibasova, Serik N. Abdreshov, Makpal A. Yessenova, Sofia K. Imankulova, Yerkenaz N. Akhatayeva

**Affiliations:** 1 Laboratory of Physiology Lymphatic System, Institute of Genetics and Physiology SC MSHE RK, Almaty 050060, Kazakhstan; khanturin55@gmail.com (M.R.K.); georgiidemchenko@mail.ru (G.A.D.); snabdreshov@mail.ru (S.N.A.); esenova_makpal@mail.ru (M.A.Y.); 2 Department of Biology, Abai Kazakh National Pedagogical University, Almaty 050060, Kazakhstan; sofia_professor@mail.ru (S.K.I.); erkenaz_akhataeva@mail.ru (Y.N.A.)

**Keywords:** aging mechanisms, lymphatic flow, interstitial humoral transport, phytotherapy, age-related pathologies

## Abstract

Aging is a multifactorial biological process characterized by a progressive decline in functional capacity at the molecular, cellular, tissue, and organismal levels. The aim of this review is to summarize current concepts of aging mechanisms and the prevention of age-related pathologies from a lymphological perspective, taking into account contemporary literature data and the results of our own studies. Currently, two major concepts dominate in gerontology: programmed aging, which considers aging as a genetically determined process, and damage accumulation theories, which associate aging with the progressive accumulation of molecular and cellular damage. The hallmarks of aging include genomic instability, telomere shortening, epigenetic alterations, impaired proteostasis and macroautophagy, dysregulation of nutrient sensing, mitochondrial dysfunction, cellular senescence, stem cell exhaustion, altered intercellular communication, chronic inflammation, dysbiosis, hormonal imbalance, and disturbances in interstitial humoral transport and lymphatic outflow. At the tissue and organ levels, impairment of lymphatic drainage is of particular importance, leading to interstitial fluid stagnation and the accumulation of toxic metabolites, which exacerbate cellular and subcellular dysfunction. Phytotherapeutic agents containing flavonoids, phenolic compounds, terpenoids, glycosides, polysaccharides, and other biologically active substances exhibit antioxidant, anti-inflammatory, and cytoprotective properties. They inhibit lipid peroxidation, reduce excessive nitric oxide production, and contribute to the restoration of interstitial humoral transport and lymphatic outflow. Interstitial humoral transport and the lymphatic system, together with renal mechanisms, play key roles in maintaining body fluid homeostasis. Targeted regulation of lymphatic system function may help reduce tissue congestion, maintain physiological homeostasis, and improve quality of life in the elderly.

## Simple Summary

Aging is a natural process that leads to a gradual decline in body functions and increases the risk of chronic diseases. While many studies focus on genetic and molecular mechanisms of aging, the role of the lymphatic system and interstitial fluid transport remains less explored. Impaired lymphatic drainage can cause the accumulation of metabolic waste, tissue congestion, and chronic inflammation, which may accelerate aging. Plant-based compounds (phytocomplexes) contain biologically active substances with antioxidant and anti-inflammatory properties. These compounds may support lymphatic function, improve fluid drainage, and help maintain tissue homeostasis. This review highlights the importance of the lymphatic system in aging and suggests that phytotherapy may be a promising approach for the prevention of age-related diseases and improving quality of life in older adults.

## 1. Introduction

Aging is a complex biological process associated with the development of degenerative changes in all body systems, including mitochondrial dysfunction, telomere shortening, epigenetic changes, cellular senescence, impaired proteostasis, genomic instability, metabolic disorders, stem cell depletion, and immune senescence [1,2,3,4]. According to researchers, it is impossible to isolate any single factor that ensures lifespan extension. Longevity is determined by a “complex interaction of hereditary and environmental factors” [5,6].

Twin studies have shown that approximately 20–30% of the differences in human lifespan can be attributed to genetics, while the remainder is due to individual behavior and environmental factors that can be modified [7].

Discovering the key links in the aging process is one of the primary goals in the development of geriatrics. In recent years, increasing research has been devoted to studying geroprotective mechanisms and the impact of various conditions and diseases on aging in general. Of particular importance is identifying age-related involutional processes in the human body and determining whether they are part of normal aging or a pathology that requires correction in old age [8].

Aging is defined by features that meet the following three prerequisites: (1) their manifestation is age-related; (2) the acceleration of aging being associated with the experimental detection of these features; and (3) the possibility of slowing, halting, or reversing aging through therapeutic interventions. The authors propose the following twelve hallmarks of aging: genomic instability, telomere attrition, epigenetic changes, impaired proteostasis, impaired macroautophagy, impaired nutrient sensing, mitochondrial dysfunction, cellular senescence, stem cell exhaustion, impaired intercellular communication, chronic inflammation, and dysbiosis. These hallmarks are interconnected with each other and with recently proposed health hallmarks, which include the organizational features of spatial compartmentalization, the maintenance of homeostasis, and adequate stress responses [2,9,10].

Age-related changes in the body are associated with the activity of the lymphatic system, which plays an important role in the body’s water homeostasis. The mechanism for maintaining fluid homeostasis is lymphatic drainage of the body’s internal environment—its endoecological space, according to Yu. M. Levin [11,12,13].

Despite extensive research on the canonical hallmarks of aging, the role of interstitial fluid dynamics and lymphatic transport remains insufficiently integrated into existing aging models. Most current concepts focus primarily on intracellular and systemic mechanisms, while processes related to tissue drainage, metabolite clearance, and interstitial homeostasis are often underrepresented. However, growing evidence suggests that impaired lymphatic function contributes significantly to chronic inflammation, metabolic imbalance, and reduced regenerative capacity. Therefore, incorporating a lymphological perspective may provide a more comprehensive understanding of aging mechanisms and open new avenues for preventive and therapeutic strategies.

Data on how to achieve a long life without the development of age-related pathologies is still limited. In this context, the concept of healthy aging is particularly relevant, with the use of medicinal plants and a rational and balanced diet recognized as key factors for maintaining health and prolonging life. According to several studies, functional foods and medicinal plants represent a promising approach to the prevention and mitigation of age-related changes. A growing body of scientific evidence confirms that bioactive compounds contained in medicinal plants have significant potential for the prevention and treatment of age-associated pathologies, including inflammatory, metabolic, and neurodegenerative diseases [14,15]. Traditional research has primarily focused on general mechanisms such as oxidative stress, inflammation, and genomic instability. However, emerging evidence highlights the lymphatic system as an important regulator of tissue homeostasis and detoxification processes. Impairment of lymphatic drainage contributes to chronic inflammation and reduced regenerative capacity—key factors in aging.

The purpose of this review is to summarize research data on theories of aging and the prevention of age-related pathologies from a lymphological perspective, as well as our own research.

## 2. Intracellular Mechanisms Associated with Aging

Proper genome function is the most important prerequisite for the stable functioning of cells and the organism as a whole. Genetic instability results from point mutations, deletions, translocations, telomere shortening, single- and double-strand DNA breaks (with subsequent incomplete repair), chromosomal rearrangements, defects in cellular architecture, and gene disruption due to viral and transposon interference [16]. Comparative analysis shows that the rate at which mutations accumulate in the cells of various mammals inversely correlates with their lifespan [10].

At the genetic level, telomerase DNA appears to be the most important factor in the mechanisms of aging. Telomere DNA undergoes natural terminal erosion through various processes, including the linear chromosomal DNA replication mechanism, which shortens telomeres with each cell division [17], as well as processes associated with nuclease action, DNA replication, recombination, and oxidative stress. Although the enzyme telomerase is capable of counteracting telomere shortening, its activity is typically absent in normal adult cells. When telomere length decreases to a critical value, it leads to replicative senescence and cell death [10,18]. A genetic predisposition to longer telomeres has been found to be associated with an increased risk of cancer [19,20].

Several independent studies have shown that shortening of telomere DNA in humans is associated with a wide range of diseases and for several age groups of patients, where it can predict future risks and outcomes, including mortality. In older people, telomere shortening occurs in all blood cells, but the rate of shortening varies among individuals. Telomere length changes with age are not stable. For example, the most dramatic decrease in length in lymphocytes occurs during the first year of life; then, this process slows down and reaches a constant level after approximately 30 years of age [21].

There is evidence demonstrating the role of stress and its influence on the rapid shortening of telomeres in cells, and this effect depends on the duration and intensity of stress [22]. For example, oxidative stress has been shown to shorten telomere length, regardless of the cause of the stress [23]. Changes in telomere length have been shown to be associated with the development of cardiovascular and infectious diseases. The risk of mortality from cardiovascular disease in patients with shorter telomeres in blood leukocytes is increased by 3 times, and that from infectious diseases by 8 times [24].

The enzyme telomerase lengthens telomeres in gametes and stem cells [25]. In somatic cells, the gene responsible for telomerase production is deactivated as a defense against the development of cancer cells. Low telomerase activity in telomerase-deficient human cells leads to a number of age-related diseases associated with the loss of tissue regenerative capacity [26]. It has been experimentally shown that accelerated aging in telomerase-deficient mice returns to normal when telomerase is reactivated [27]. Moreover, normal aging in mice can be delayed by pharmacological activation or transduction of telomerase, and mice with ultra-long telomeres exhibit extended lifespan and improved metabolic health [26,28].

Another method involves the analysis of epigenetic mechanisms regulating gene expression—DNA methylation. DNA methylation occurs through the addition of a methyl group (CH3-) to the nitrogenous base of the nucleotide cytosine, thus suppressing gene expression and preventing RNA synthesis. Widely used DNA methylation age predictors were described by Horvath and Hannum [29], a method later dubbed the “epigenetic clock” (DNAmAges). A 5-year increase in the “epigenetic age” determined by Horvath’s method increased the risk of death by 22%, while such an increase determined using Hannum’s method increased the risk by 16% [30]. A clear association between longevity and a slowing of the “epigenetic clock” was also demonstrated in Italian centenarians [31].

**Figure 1:**
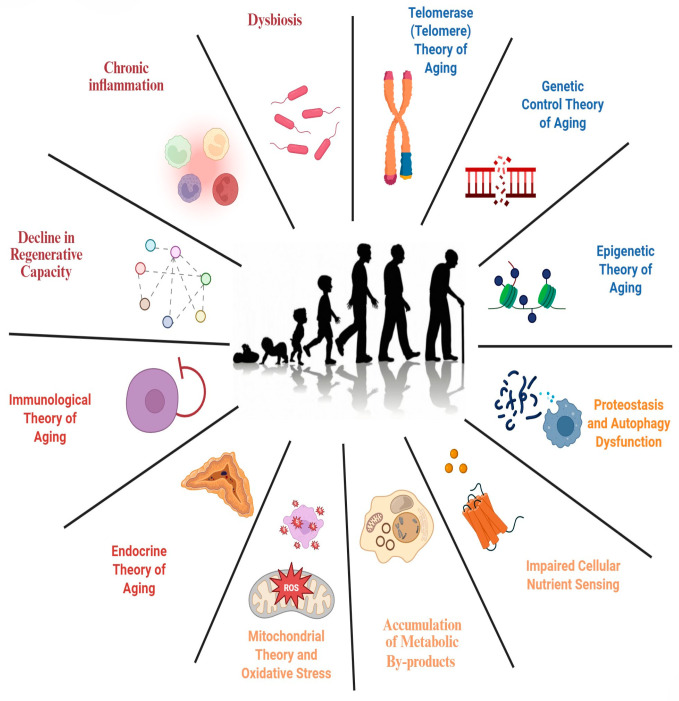
Main mechanisms of aging. Aging is driven by interconnected processes, including telomere shortening, genetic and epigenetic alterations, impaired proteostasis and autophagy, mitochondrial dysfunction, the accumulation of metabolic byproducts, oxidative stress, chronic inflammation, and microbiota dysbiosis. These processes reduce the regenerative potential of cells and tissues and collectively lead to the gradual functional decline of the organism. The figure illustrates the integrative interplay among all mechanisms of aging.

With age, mitochondrial efficiency tends to decline. The reasons for this are not yet fully understood, but several mechanisms are suspected: the accumulation of damage and mutations in mitochondrial DNA, the oxidation of mitochondrial proteins, and failures in quality control during mitophagy [32]. The key intracellular process in maintaining mitochondrial quality is mitophagy, which prevents the accumulation of defective mitochondria within the cell. Mitophagy deteriorates with age, accelerating aging and being a serious risk factor for many diseases, including neurodegenerative diseases (Figure 1) [33].

Proteostasis also plays a critical role in aging mechanisms. Proteostasis maintains a homeostatic pool of functional proteins necessary for normal cellular function [34]. Misfolded, oxidized, or improperly modified proteins can form non-functional or toxic aggregates, impair cellular function, and contribute to age-related diseases, such as amyotrophic lateral sclerosis, Alzheimer’s disease, Parkinson’s disease, and cataracts [35,36]. Autophagy, the process by which cells deliver their own components—including proteins and organelles—to lysosomes for degradation, is often impaired with aging. This process provides energy, removes damaged or unnecessary components, and eliminates intracellular pathogens. Dysfunctional autophagy is associated with several age-related conditions, including neurodegeneration and immunosenescence [37]. In a study of long-lived individuals, CD4+ lymphocytes from 16 descendants of centenarians exhibited higher autophagic activity compared to age-matched controls [10]. Age-related diseases are also associated with impaired nutrient-sensing mechanisms. Cellular nutrient sensing allows cells to detect changes in nutrient availability and respond appropriately. Nutrients such as glucose, fatty acids, and amino acids regulate anabolic pathways, among which the mechanistic target of rapamycin (mTOR) pathway is well studied. Under nutrient deprivation, AMP-activated protein kinase (AMPK) inhibits mTOR to conserve resources [38].

Loss of regenerative capacity is one of the most evident consequences of aging. This decline is primarily due to the gradual reduction in the number and proliferative capacity of stem cells. For example, reduced hematopoiesis leads to a decrease in immune cell numbers, increased risk of anemia, and higher susceptibility to myeloid malignancies. Stem cell rejuvenation has been shown to reverse some manifestations of aging at the organismal level [39]. The transplantation of stem cells derived from young mice into progeroid mice increased lifespan and mitigated tissue degeneration [2].

## 3. The Importance of Reactive Oxygen Species in the Mechanisms of Premature Aging

The role of reactive oxygen species as the primary source of damage to cellular components during aging was proposed by D. Harman in 1956 [40]. Reducing the amount of reactive oxygen species using antioxidants formed the basis of V.P. Skulachev’s proposed method for combating age-related diseases [41]. During the process, the oxygen molecule is reduced, sequentially passing through a series of intermediate states, such as the superoxide radical (O^2−^) and hydrogen peroxide. In most cases, these molecules, called reactive oxygen species, remain bound to cytochrome c oxidase until the oxygen and hydrogen are completely converted to water. Unlike the orderly reduction of oxygen by cytochrome c oxidase, oxygen molecules can sometimes interact randomly with reduced components of the respiratory chain during electron transfer and transform into highly reactive superoxide radicals. With age, there is a significant decrease in endothelium-dependent vasorelaxants (primarily NO) [42]. It is believed that the balance between vasoconstrictors and vasodilators is also disrupted.

**Figure 2:**
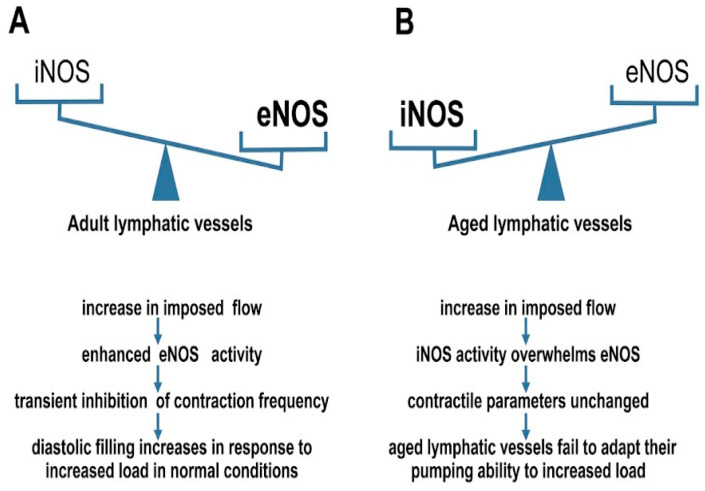
NO-dependent regulatory mechanisms in aged lymphatic vessels. (**A**) In the lymphatic vessels of adults, increased activity of eNOS (endothelial nitric oxide synthase) leads to the production of NO (nitric oxide), which mediates a temporary reduction in the contraction frequency of lymphatic vessels in response to increased applied flow. This allows the vessels to adapt to the load and maintain optimal lymphatic flow. (**B**) In aging lymphatic vessels, elevated levels of iNOS (inducible nitric oxide synthase) result in sustained NO levels, so the contraction parameters of the lymphatic vessels do not change in response to increased applied flow, reflecting a reduced adaptive capacity.

Endothelial NO synthase (eNOS) is activated by “endothelium-dependent vasodilators”, namely, surface receptors for acetylcholine, bradykinin, serotonin, nucleotides, and thrombin released from platelets. NO activates soluble guanylate cyclase, thereby stimulating the synthesis of cyclic guanosine monophosphate (cGMP), leading to a decrease in cellular Ca^2+^ concentration and relaxation of smooth muscle cells. In the absence of cofactors, eNOS can be a source of superoxide (O^2−^), which is the main oxidizer for NO [43]. Thus, Figure 2 reflects age-related changes in lymphatic vessels and the mechanisms of eNOS activation, including chemical and mechanical stimuli that affect vascular function.

There is a causal relationship between increased O^2−^ production and decreased NO bioavailability. O^2−^, when interacting with NO, rapidly forms the toxic radical peroxynitrite (ONOO^−^). The latter readily penetrates the cell membrane and initiates the oxidative modification (nitrosylation) of certain proteins, leading to irreversible cellular damage. Furthermore, ONOO^−^ triggers endothelial cell apoptosis, which inevitably leads to inflammatory and immune responses in the vascular wall. These reactions impair vasorelaxation in aging vessels and subsequently lead to platelet adhesion and aggregation on the arterial surface and activation of medial smooth muscle cells’ proliferation, followed by their migration into the intima. This is considered a pathogenetic link in age-associated atherosclerosis and arterial hypertension [44].

Thrombosis may occur in the lymphatic vessels, and varicosity of lymphatic collectors is observed. Sclerotic changes develop in the vessel walls. All of the above often lead to a weakening of the lymphatic system’s drainage function, which in turn enhances inflammatory processes in the tissues [13].

## 4. The Role of Inflammatory Stress in Aging Theory

One of the key aspects of human aging is the development of low-grade chronic inflammation, or inflammatory aging. A large body of experimental and epidemiological data suggests a negative impact of inflammatory aging on physical and cognitive performance, as well as lifespan. The mechanisms underlying inflammatory aging are diverse and include the accumulation of senescent cells, epigenetic disruption, increased permeability of epithelial barriers to immune-stimulating microbial substances, the excessive release of endogenous danger signals, persistent infections, and impaired mechanisms for resolving acute inflammation [45].

The main manifestation of inflammatory aging is an increase in the basal production of pro-inflammatory cytokines and a number of other inflammatory mediators in old age, leading to increased plasma levels [46]. Macrophages were initially considered the “culprits” of inflammatory aging. According to modern concepts, the cellular sources of inflammatory aging are much more diverse, although macrophages and monocytes still play the main roles [47].

The lymphatic system plays a crucial role in the modulation of inflammatory stress. Lymphatic vessels and lymph nodes enable the drainage of cytokines and other inflammatory mediators, thereby maintaining local and systemic homeostatic balance. With aging, lymphatic function declines, which promotes the accumulation of pro-inflammatory factors and contributes to the transition of inflammation to a chronic state.

## 5. Dysbiosis

With age, the gut microbiome undergoes significant changes, primarily a shift in the abundance of various microorganisms and a decrease in their diversity [48]. Combined with the loss of structural integrity of the intestine and other barriers (such as the blood–brain barrier), these changes in microbial populations may contribute to chronic inflammation [9]. In this context, the lymphatic system plays a key role: through Peyer’s patches, mesenteric lymphatic vessels, and lymph nodes, products of microbial metabolism, bacterial antigens, and cytokines are transported from the intestine to the systemic circulation, thereby modulating the immune response. With advancing age, lymphatic function declines, leading to the accumulation of pro-inflammatory factors and contributing to a persistent inflammatory state. Given the influence of the gut microbiome on the host, it is conceivable that altering this microbiome may improve lifespan and quality of life. Recent research has demonstrated that microbiomes are crucial factors in many physiological processes, such as the digestion and absorption of nutrients, protection against pathogens, and the synthesis of essential metabolites, including vitamins, amino acids, secondary bile acids, and short-chain fatty acids. The gut microbiota also sends signals to the peripheral and central nervous systems and other distant organs, thereby strongly influencing the overall health of the host [49]. Disruptions in bidirectional host–bacterium communication lead to dysbiosis and contribute to the development of various pathological conditions, such as obesity, type 2 diabetes, ulcerative colitis, neurological disorders, cardiovascular disease, and cancer [49].

The transplantation of gut microbiota from old to young mice induces an inflammatory response characterized by increased CD4+ T-lymphocyte differentiation in the spleen, upregulation of inflammatory cytokines, and increased circulation of bacterial-derived inflammatory factors [50]. These effects are mediated, at least in part, by the lymphatic system, which facilitates the transport of microbial metabolites and antigens to the lymph nodes, where they are recognized by immune cells. FMT also provides evidence that the gut microbiome is involved in maintaining brain health and immunity during aging [51]. These and other studies demonstrate a causal relationship between dysbiosis and the rate and quality of aging. Research suggests that restoring youthful microbiota may extend the duration of maximum and healthy lifespan [10].

## 6. The Endocrine Theory of Aging

The founder of the endocrine theory of aging, French pathophysiologist Charles-Édouard Brown-Séquard, defined aging in 1889 as a consequence of “a disturbance in the hormonal secretion of the body at the holistic physiological level of protoplasmic organization” [52]. Today, there is reliable data showing that endocrine gland activity changes at different stages of ontogenesis. For example, elderly patients (≥60 years) are more often found to have subclinical hypothyroidism, with thyroid-stimulating hormone levels in the range of 7–10 mlU/L [53], decreased total and free testosterone fractions [54], and a marked decrease in peak melatonin concentrations by an average of 50% compared to levels at age 20 [55].

Another illustrative example of endocrine aging is menopause (in women) and andropause (in men) [56,57]. The onset of this period in women is associated with a combined decrease in testicular and ovarian function, accompanied by laboratory signs of hypergonadotropic hypogonadism; insufficient secretion of growth hormone and somatomedins (insulin-like growth factor (IGF-1, -2)); hyperreactivity of the adrenal medulla; and increased activity of nuclear mineralocorticoid receptors with the development of metabolic (obesity and type 2 diabetes mellitus), cardiovascular (endothelial dysfunction and dyslipidemia), musculoskeletal (osteopenia, osteoporosis, and sarcopenia), and urogenital disorders (genitourinary syndrome, urinary incontinence, and dysuria) [56].

The most common thyroid pathology in old age is subclinical hypothyroidism, characterized by a persistent deficiency of thyroid hormones [8,58].

In old age, lipid metabolism plays an important role in achieving compensation for many diseases, especially cardiovascular diseases. Subclinical hypothyroidism is characterized by elevated levels of lipoprotein(a), which contains the apolipoprotein-A molecule linked to apolipoprotein-B-100, a component of very-low-density lipoproteins, giving lipoprotein(a) atherogenic and thrombogenic properties [59].

In a prospective population-based cohort study, L. Chaker et al. analyzed TSH and fT4 levels among participants over 65 years of age. Data analysis revealed that higher TSH levels and low fT4 levels are associated with a higher risk of developing type 2 diabetes mellitus and progression from prediabetes to diabetes [60].

Thus, it is important to evaluate thyroid function in patients with various carbohydrate metabolism disorders. Achieving a euthyroid state in this group can improve the prognosis of the disease. One condition that requires particular care in the treatment of patients with hypothyroidism is coronary artery disease (CAD). It is believed that hypothyroidism, due to its atherogenic effect, contributes to the development and progression of CAD [57].

A study by N. Rodondi et al. assessed the risk of developing CAD in patients with subclinical hypothyroidism. Data from 25,977 patients were used for the evaluation, obtained from the MEDLINE and EMBASE databases (from 1950 to 31 May 2010). The results showed that subclinical hypothyroidism was associated with an increased risk of developing CAD, especially in patients with TSH concentrations of 10 mU/L or higher [61].

## 7. The Importance of Metabolic Byproducts

The next theory explains aging as the accumulation of various types of chemical substances that cannot be processed by the body. This theory has been well formulated in a number of works by V. Gladyshev. It is proposed that, due to the fundamental imperfections of chemical and, in particular, enzymatic transformations, side reactions occur in cells during metabolism. The more complex and intense the metabolism, the more the types of byproducts that appear in cells. Some of these products easily leave the cells, while others are utilized by specialized enzymes. Each byproduct requires its own enzyme, or possibly several, to utilize it, which, in turn, further complicates metabolism and leads to an even greater diversity of byproducts [62,63].

Unutilized metabolic byproducts accumulate not only inside but also outside cells. Among the extracellular products characteristic of humans, we can mention plaques that accumulate in blood vessels, containing cholesterol and its oxidation products, as well as polymerized protein fragments, such as ß-amyloid in nervous tissue. Atherosclerotic plaques are composed of lipids deposited on the walls of blood vessels. These primarily consist of oxidized and glycosylated forms of cholesterol, but other lipids are also present. The main carrier of lipids in the bloodstream is low-density lipoproteins, from which lipids reach the vessel walls. Immune system cells—monocytes—interact with atherosclerotic plaques, transforming into macrophages. These cells ingest the plaque contents and, up to a certain point, play a positive role. However, in some cases, the macrophages themselves begin to accumulate in the plaque material, forming lipid-filled foam cells [64].

It should be noted that metabolic byproducts can only be removed from the interstitial space by the lymphatic system, whose normal functioning ensures their timely elimination [65].

The second type of toxic product, accumulating primarily in nervous tissue, is amyloid proteins. The most well-known is the so-called β-amyloid, which causes Alzheimer’s disease. It is formed from a protein called β-amyloid precursor, resulting from the excision of the central portion of the precursor by β- and γ-secretases [65].

The mechanisms of damage associated with normal metabolism are also interconnected. Primarily, these are the formation of reactive oxygen species, leading to the oxidation of cellular components, and the glycation of cellular components associated with the main metabolite, glucose. The products of oxidative damage and glycation lead to the deposition of lipofuscin in lysosomes and decreased vascular elasticity, and contribute to the deposition of insoluble products both on vascular walls and in nervous tissue [66].

## 8. The Importance of Interstitial Transport and Lymph Flow in Aging Mechanisms

According to another theory of aging, its main cause is intoxication of the body due to difficulty removing accumulated metabolic products from the interstitium [67]. Given that endogenous intoxication increases with age, it is believed that aging is intoxication [68]. Both age and intoxication cause unidirectional disturbances in various parts of the microcirculatory system, both circulatory and lymphatic [69,70,71].

Age-related changes in the body are associated with the activity of the lymphatic system, which plays an important role in the body’s water homeostasis. The mechanism for maintaining water homeostasis is lymphatic drainage of the body’s internal environment—its endoecological space, in the terminology of Yu. M. Levin [11,12,13]. Lymphatic drainage is combined with the neutralization of harmful substances present to a greater or lesser extent in the interstitium surrounding the cell. Yu. I. Borodin proposed calling this process natural intracorporeal lymphodetoxification [72,73]. Three homeostatic systems participate in the neutralization of substances harmful to the body’s vital functions—the lymphatic, lymphoid (immune), and loose connective tissue systems—which form the interstitium: the body’s internal environment. This functional synergism provides grounds for considering these systems as a drainage–detoxification complex. Extrapolating this concept to the body level, we should consider the drainage–detoxification complex as a functional system that ensures the body’s biosafety [74].

When examining the aging process from a lymphological perspective, it is impossible to ignore the issue of optimal hydration. Different authors cite different values for optimal water content in human tissues depending on age. According to some scientists, natural death occurs due to tissue dehydration. Cells are deprived of their aqueous habitat, intracellular metabolism is disrupted due to a lack of external supply of energy and plastic materials, and harmful metabolites are not completely eliminated from the cell and accumulate in the surrounding interstitium [75,76,77].

This scenario was apparently first proposed by I. I. Mechnikov [78]. He asserted that death occurs due to the body’s self-poisoning. I. I. Mechnikov believed that aging is based on the process of “hysteresis”, i.e., the slow removal of harmful metabolites from the cell. This view is also shared by other scientists [79,80].

There is a rough scale for the degree of tissue hydration in different periods of life. Masaru Emoto suggests the following gradation: fetal water saturation—99%; newborn—90%; adult—70%; and old age—50%. Moreover, the latter value predetermines a person’s natural death [81]. Different authors cite different values for the optimal water content in human tissues depending on age. Most scientists agree only that tissue hydration decreases with age. However, not everyone agrees with this. Visser et al. found no difference in tissue hydration when studying people aged 20 to 94 years [82].

The movement of lymph is facilitated by the presence of lymphangions in the lymphatic vessel—intervalvular segments of the lymphatic vessels that have their own contractile activity and promote lymph movement in a centripetal direction and, ultimately, into the circulatory system, providing tissue drainage [83,84]. In our studies, we observed a decrease in interstitial fluid volume in laboratory rats with age, by 11% compared to adulthood and by 20% compared to old age. Blood plasma volume also decreased by 6.25% and 8.4%, respectively. Lymph flow decreased by 40% in adulthood and by 64% in old age. Blood and lymph viscosity increased by 18% from young to old animals. All of this indicates that tissue hydration decreases with age [85,86].

Involutional changes in the lymphatic system led to impaired contractile function of the lymphatic vessels and nodes; for example, inhibition of contractile activity of the cervical and mesenteric lymph nodes was observed with age [86,87,88].

The degree to which initial lymphatics are filled with tissue fluid determines the magnitude of lymph formation and lymph flow. Loose connective tissue also has a close functional relationship with the lymphoid (immune) system, as the interstitium contains a greater or lesser number of immunocompetent cells (tissue lymphocytes), which locally form permanent or temporary lymphoid nodules. It follows from the above that it is impossible to examine the age-related evolution and aging of the lymphatic system in isolation without considering processes in the interstitium and lymphoid structures [89]. All three homeostatic systems function and age as part of a single drainage–detoxification complex—the body’s protective system. Indeed, involutional changes in the lymphatic system—in the vessels and lymph nodes—are combined with signs of aging in the loose connective tissue and the lymphoid (immune) system. The most general (universal) signs of aging are found in connective tissue. With aging, the mass of collagen fibers increases, and their resistance to collagenase, trypsin, and pepsin increases. “Collagenization” of reticulin fibers occurs, and coarsening of elastic fibers is also noted. Their sensitivity to trypsin and elastase increases [90]. These structural changes in loose connective tissue are associated with increasing tissue hypoxia, an increase in the number of free radicals, a shift in the pro- and antioxidant balance toward the acidic side, and an increase in the number of lipid peroxidation products. These biochemical changes in the interstitium disrupt the normal process of lymph formation and lymph flow [91]. Thus, aging is associated with a deficiency in interstitial transport and lymphatic drainage.

When considering the problem of aging, it is necessary to consider the close functional connections between three homeostatic systems: lymphatic, lymphoid, and loose connective tissue systems. The latter forms the internal environment of the body. The main colloid-like substance of loose connective tissue, which forms the body’s interstitial space, is reinforced with bundles of collagen fibers. The direction of the fibrous basis of the interstitium is such that channel-like spaces can be traced along the bundles, which are natural pathways for the movement of mobile water and dissolved or suspended substances of endo- or exogenous origin. These tissue gaps are pathways for non-vascular tissue microcirculation and are functionally connected to the roots of the lymphatic system, hence their name—initial lymphatics [92].

The degree of filling of the initial lymphatics with tissue fluid determines the magnitude of lymph formation and lymph flow. Thus, initial lymphatics are considered the first link in the lymphatic drainage mechanism.

Structural changes in loose connective tissue are associated with increasing tissue hypoxia, an increase in free radicals, a shift in the pro- and antioxidant balance toward the acidic side, and an increase in the amount of lipid peroxidation products. These biochemical changes in the interstitium disrupt the normal process of lymph formation and lymph flow.

The lymphatic capillary networks of an aging organism, embedded in the connective tissue framework of an organ, become deformed due to the deformation of the architecture of the surrounding connective tissue. A reduction in some organ lymphatic networks is observed, with the appearance of lymphatic lacunae and microcysts that lose their connection with the vascular network. The remaining lymphatic vessels appear narrow and tortuous, and characteristic angularity appears. Not only is lymph formation impaired, but lymph flow from the organ is also reduced. Along with the depletion of the organ lymphatic bed due to partial reduction in the vascular network, areas of lymphostasis arise, with the formation of organ or extraorgan lymphatic cavities (cysts), which are difficult to diagnose and treat. Fibrosis with atrophy and even rupture of the lymphatic vessel wall has been noted. Thrombus formation is possible in the vessels, varicose veins of the lymphatic collectors are observed, and sclerosis develops in the vessel walls [93].

Ultrastructural, biochemical, and proteomic analyses indicate a loss of matrix proteins and smooth muscle cells in aged lymphatic collectors, resulting in decreased contraction rate, systolic lymph flow velocity, and pumping activity of lymphatic collectors; decreased endothelial cell glycocalyx thickness; and loss of gap junction proteins in aged lymphatic collectors. Redox proteomic analysis revealed an aging-associated increase in the glycation and carboxylation of proteins in endothelial cells and the lymphatic matrix. Functionally, these changes result in apparent increased lymphatic vessel permeability, allowing pathogen penetration from the collectors into surrounding tissues, and a decreased ability to control tissue fluid homeostasis. These changes disrupt lymph flow, disrupting tissue fluid homeostasis and pathogen transport [87].

Lymph nodes are peripheral organs of the immune system, located along the lymphatic drainage pathway from organs and body parts. Lymph nodes perform barrier-filtration, cytopoietic, and immunopoietic functions. In the lymph nodes, located along the lymphatic vessels and serving as biological and mechanical filters for the lymph flowing through them [94,95], lymph undergoes detoxification, which is carried out at three levels: (1) biophysical processes (absorption, filtration, etc., in the reticular tissue); (2) biochemical transformation (lysozyme, the complement system, tumor necrosis factor, enzymes—monoamine oxidase (MAO), cytochrome P-450, etc.); (3) immunobiological processing (cellular and humoral immune response). Lymph nodes are histologically and functionally divided into three zones: the cortex, the paracortical zone, and the medulla. The cortex of the lymph node is a B-dependent zone consisting of primary and secondary lymph nodes (follicles). The proliferation center is mainly represented by B-lymphocytes at various levels of differentiation and a small number of dendritic cells of the reticular stroma. The paracortical zone is a T-dependent zone, surrounds the follicles of the cortex, and is located between it and the medulla. The medulla of the lymph node is formed by small B-lymphocytes organized into medullary cords, between which are the medullary sinuses [96]. According to various sources, the human body contains 400 to 800 lymph nodes. The general rule of lymph flow is that lymph, while moving from its site of formation to its entry into the large veins of the neck, must pass through at least one lymph node (for example, lymph formed in the wall of the stomach passes through 6 lymph nodes) [97]. Under the capsule of the lymph node is the subcapsular sinus, a key structure that determines the early response of the lymphatic system to an incoming antigen. Antigens, cytokines, and other molecules transported by the lymph contact these cells, thereby triggering immune processes in the lymph node. Trabeculae, which are the supporting structures of the lymph nodes, extend from the capsule into the node. The space between the trabeculae contains lymphoid tissue organized into follicles. The proliferation and differentiation of B-lymphocytes occur in the area adjacent to the capsule [98]. Below, in the paracortical zone, T-lymphocytes predominate, undergoing antigen-dependent proliferation and differentiation. The lymph node performs several important functions, including acting as a mechanical and biological filter. Bacteria, viruses, and cancer cells are trapped in the lymph node’s reticular structure and analyzed by antigen-presenting cells [99].

The immune (lymphoid) system undergoes changes synchronous with the lymphatic system in old age. These changes are clearly evident in the structure of the lymph nodes. Summarizing the aging process of the lymph nodes, it should be noted that, in old age, lymphoid tissue undergoes involution, being replaced to a greater or lesser extent by connective or adipose tissue. The reticular tissue inherent to the lymph nodes undergoes coarsening, partially transforming into bundles of collagen fibers, and the cellular composition of the lymphoid tissue also changes. The total number of lymphoid cells decreases [100,101]; when they decrease, the frequency of infectious, autoimmune, and tumor diseases increases.

Structural changes that occur with age affect the functioning of immunocompetent cells, which can ultimately lead to less effective or reduced immune responses. (1) The principle of regional determinants is crucial in the formation of the microelement and morpho-immune status of lymph nodes in different locations during aging, according to the concept of the lymphatic region. (2) The microelement profile changes with aging depending on the location of the lymph nodes that have undergone age-related changes. The degree of accumulation of each microelement varies in regional lymph nodes, leading to the formation of an individual microelement profile. (3) The morpho-immune status of regional lymph nodes varies with aging and is determined by the representation of compartments necessary to ensure an immune response of the humoral type in the mesenteric lymph node, a mixed type in the tracheobronchial lymph node, and a cellular type in the inguinal lymph node (regional specificity). (4) The microelement profile and lymph node morphotype are interconnected, as the accumulation characteristics of each microelement influence lymphoid cell proliferation, compartment development, and the morphotype of lymph nodes that have undergone age-related changes [102,103].

As can be seen, with age, all three homeostatic systems—the lymphatic, lymphoid, and loose connective tissue systems—synchronously decrease their functional capacity, thereby paving the way for the development of diseases common in the elderly: atherosclerosis, cancer, and diabetes.

Therefore, preventative and restorative lymphology methods should be included in measures aimed at preventing and overcoming age-related diseases. These methods include lymphatic stimulation, lymphoprotection, and lymphatic correction, sometimes combined into the concept of lymphotropic therapy. It is noted that all general anti-sclerotic measures slow the aging process, as all three systems of the drainage–detoxification complex are subject to sclerosis. Changes occur in the pericellular humoral transport system, limiting both the delivery of essential nutrients, drugs, and oxygen to the intercellular matrix and then into the cell, and the removal of metabolic products [104,105,106,107].

Accumulating in the pericellular space, metabolites and xenobiotics can trigger a complex of reactions, such as the stimulation of phagocytosis and cytokine production, and the activation of lipid peroxidation, the kallikrein–kinin system, complement, blood coagulation, and lymphatic drainage. Progressive intoxication leads to decreased activity of oxidative enzymes and a decline in the ability to adapt to decreased blood oxygen tension, resulting in the development of hypoxic, circulatory, and tissue hypoxia [108]. These changes become a prerequisite for premature aging and predetermine age-specific disease progression—polymorbidity, a tendency toward chronicity, the presence of complications, and the need for a large number of medications. Consequently, elderly and senile individuals, as well as patients with endogenous and exogenous intoxication, regardless of their underlying pathology, require antioxidant protection and enhanced humoral transport in the intercellular matrix and lymphatic drainage.

The idea of the possibility of drug-mediated control of interstitial humoral transport and lymphatic drainage of tissues, put forward by Yu. M. Levin et al., opens new avenues for solving this problem [12].

## 9. Aging of Lymphatic Endothelium, Inflammatory Signaling, and Key Intracellular Pathways

With age, the functions of lymphatic vessels and their endothelium undergo substantial alterations, associated with both cellular processes and systemic mechanisms of aging. Endothelial cells of aging lymphatic vessels exhibit reduced barrier function, diminished contractile activity of lymphatic collectors, and enhanced local inflammation, which impairs the maintenance of tissue homeostasis and promotes the accumulation of pro-inflammatory mediators throughout the body. Studies indicate that aging affects not only the morphology of lymphatic vessels but also the expression of cellular senescence markers, vascular network density, and immune cell migration via lymphatic pathways, thereby amplifying tissue inflammation [109,110].

At the molecular level, age-related changes in lymphatic endothelial cells are closely linked to dysregulation of key intracellular signaling pathways, including mTOR, AMPK, and autophagy, which are critical for maintaining cellular homeostasis. The mechanistic target of rapamycin (mTOR) pathway regulates cellular metabolism and growth; its hyperactivation during aging suppresses autophagy, leading to the accumulation of damaged organelles and protein complexes, thereby enhancing inflammatory responses and promoting the senescence phenotype [111,112]. Nuclear mTOR has been shown to mediate interactions between metabolism and epigenetic modifications, integrating nutrient signaling and gene expression programs related to growth and metabolism, thus serving as a central hub for cellular homeostasis [113].

Moreover, mTOR activity influences the senescence-associated secretory phenotype (SASP) of aging cells, which is accompanied by increased production of pro-inflammatory cytokines, including IL-6, exacerbating local and systemic inflammation [114].

In contrast, AMP-activated protein kinase (AMPK) functions as an energy sensor, regulating metabolism and stress resilience. Its activation inhibits mTOR, stimulates autophagy, and promotes the clearance of damaged cellular components, thereby protecting the endothelium from age-related dysfunction and inflammatory stress. With aging, AMPK activity declines, impairing endothelial function and increasing oxidative stress, whereas its activation can improve vascular reactivity and maintain nitric oxide synthesis [115,116].

Autophagy plays a critical role in maintaining cellular quality control, including the removal of damaged mitochondria, particularly under age-related oxidative stress [117]. Impairment of autophagic flux accelerates endothelial aging, reduces cellular adaptability, and promotes the transition to a senescent state, exacerbating local inflammation. Autophagy regulation is mediated by the interplay between AMPK and mTOR: under energy deficit, AMPK is activated, which inhibits mTORC1 and initiates autophagic processes to preserve cellular homeostasis [118].

Thus, the aging of lymphatic endothelium results from a complex interaction of metabolic signals, energy stress, and autophagy dysfunction, leading to enhanced inflammatory processes and decreased vascular adaptability. Modulation of these pathways represents a promising strategy for maintaining lymphatic function and mitigating age-related disturbances in lymphoid-immune homeostasis.

## 10. Phytotherapy for the Prevention and Treatment of Diseases Associated with Aging

Despite advances in biology and medicine, there is still a pressing need for scientific substantiation and the search for non-drug methods for the prevention and treatment of aging. The existing morphofunctional characteristics of the organs and systems of the body of an aging person largely determine the adequacy of their responses, which should be taken into account when choosing non-drug correction methods. This is especially true for the elderly and senile, whose lymphatic system structure and function, including the lymph nodes, are impaired due to the accumulation of metabolites and xenobiotics in the body. Consequently, elderly individuals require optimization of lymphatic system function at all levels, which requires increasing functional reserves and non-specific resistance. This can be achieved using non-drug correction methods, among which phytotherapy is of particular interest due to its positive effects on the body. Data on how to achieve longevity without the development of age-related pathologies are still limited. In this context, the concept of healthy aging is particularly relevant, with the use of medicinal plants and a rational and balanced diet recognized as key factors for maintaining health and prolonging life. According to a number of studies, functional foods and medicinal plants represent a promising approach to preventing and mitigating age-related changes [14,15].

Medicinal plants are a unique category of plant materials that combine nutritional value with pharmacological activity [119]. According to the World Health Organization [120], approximately two-thirds of the world’s population, especially in rural and developing regions, continue to use medicinal plants and plant-based products to treat various diseases. Their popularity is due not only to their wide availability and relative affordability but also to their perceived safety and multidisciplinary therapeutic activity. A growing body of scientific evidence confirms that bioactive compounds contained in medicinal plants have significant potential in the prevention and treatment of age-associated pathologies, including inflammatory, metabolic, and neurodegenerative diseases [15,121]. The anticarcinogenic activity of a number of plant flavonoids was reviewed in detail by Rahaman et al. [122], who noted that compounds such as quercetin, luteolin, and kaempferol exhibit antitumor properties due to their ability to inhibit cyclooxygenase-2 and matrix metalloproteinases.

**Figure 3:**
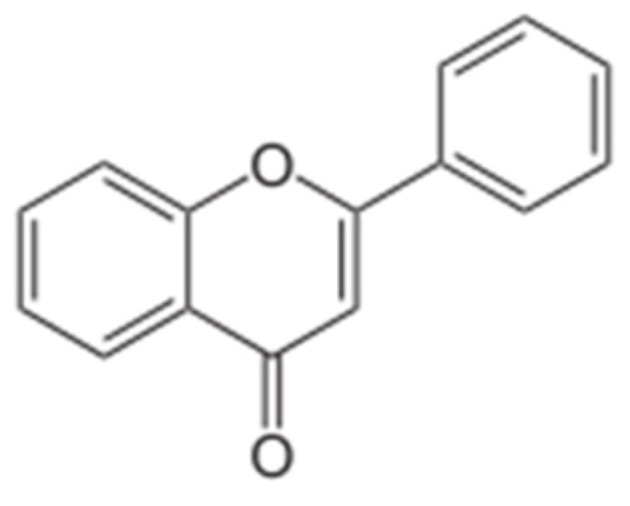
Structural formula of flavone.

The most well-known plant compounds that influence the normalization of bodily functions are flavonoids. Flavonoids are the largest class of plant polyphenols. Chemically, flavonoids are hydroxy derivatives of flavone (flavonoids proper), 2,3-dihydroflavone (flavanones), isoflavone (isoflavonoids), and 4-phenylcoumarin (neoflavonoids) (Figure 3). Flavones with a reduced carbonyl group (flavonols) are also often considered flavonoids. These compounds include other C6-C3-C6 compounds, which contain two benzene rings linked to each other by a three-carbon fragment—chalcones, dihydrochalcones, and aurones [123].

Flavonoids have proven antioxidant activity. This is apparently their primary property, protecting the body’s cells from various types of damage, including age-related damage.

**Figure 4:**
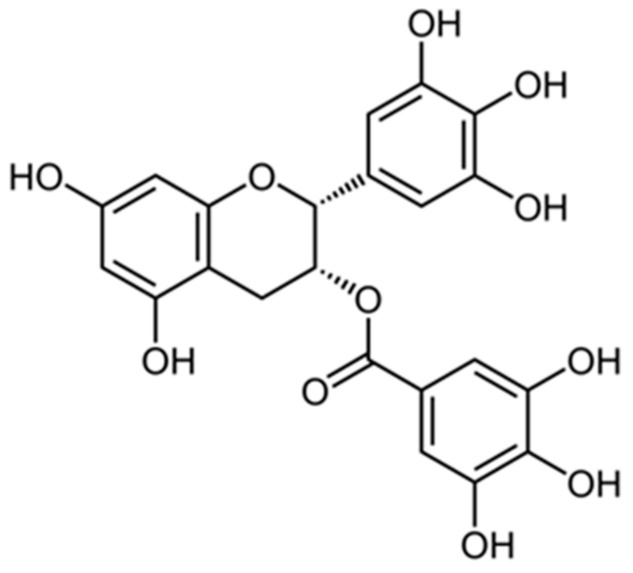
Structural formula of epigallocatechin-3-gallate.

The anticarcinogenic activity of a number of plant flavonoids was examined in detail by Rahaman et al. [124], who noted that compounds such as quercetin, luteolin, and kaempferol exhibit antitumor properties due to their ability to inhibit cyclooxygenase-2 and matrix metalloproteinases. Quercetin, hesperidin, and delphinidin are also used in flavonoid-containing compositions aimed at preventing and correcting age-related skin changes [125]. The catechin epigallocatechin-3-gallate (EGCG), a polyphenolic compound belonging to the flavonoid group, is a powerful antioxidant found in foods such as tea, apples, cocoa, strawberries, and dark chocolate (Figure 4). 

It is especially abundant in green tea, as well as oolong, pu-erh, and black tea. Its content can reach 20–25% of the dry weight in young shoots of the tea plant. It may positively influence lifespan by slowing cellular aging and protecting organs from age-related damage [126].

Flavonoids are known to influence the activity of immune cells such as macrophages and lymphocytes, and to modulate the production of pro-inflammatory cytokines, including within lymph nodes [127]. It has been shown that flavonoids affect vascular permeability and microcirculation, thereby indirectly supporting efficient lymphatic drainage [128]. 

**Figure 5:**
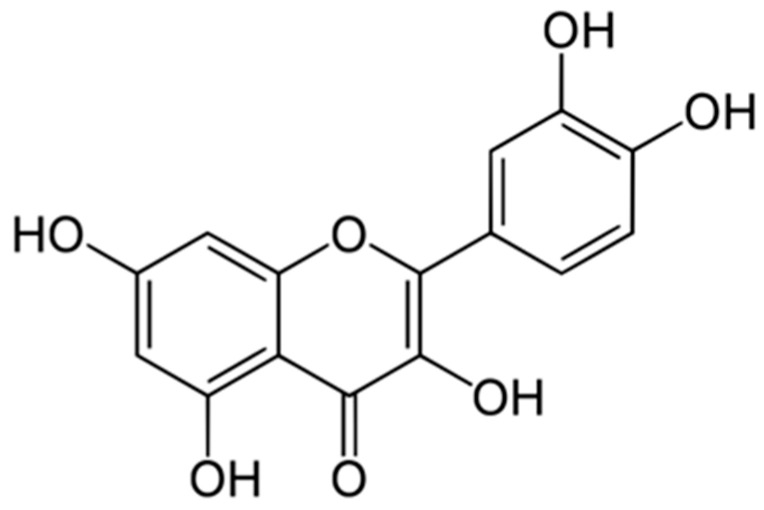
Structural formula of quercetin.

Quercetin, a natural, non-toxic flavonoid with antioxidant, anti-apoptotic, and anti-inflammatory properties in safe doses, plays a significant role in the treatment of aging-related diseases by reducing oxidative stress and inflammation and restoring mitochondrial dysfunction (Figure 5) [129,130].

In 2008, cell culture experiments demonstrated that the flavonoid quercetin is a potent antioxidant that also possesses anti-inflammatory properties. Animal model experiments demonstrated its antioxidant and anti-inflammatory effects. Quercetin has demonstrated efficacy in reducing serum levels of TNF-α, IL-1β, IL-6, and nitric oxide (NO). This process is also associated with an increased secretion of IL-10, reflecting modulation of inflammatory cytokine responses [131,132].

**Figure 6:**
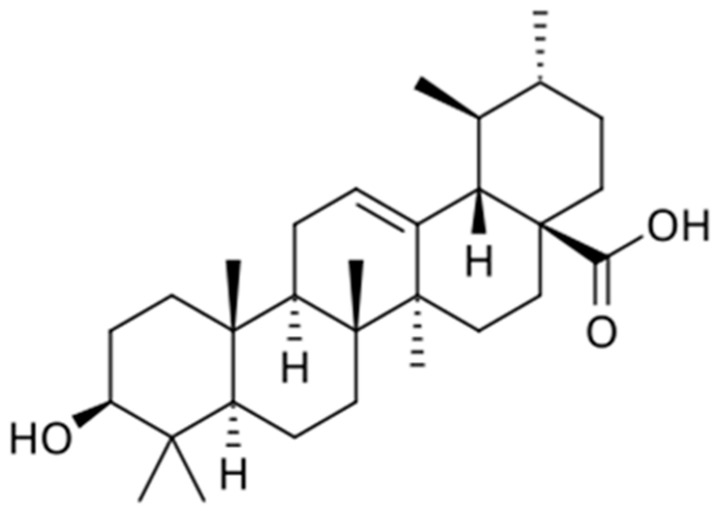
Structural formula of ursolic acid.

Ursolic acid is found in many plants, including fruit peels. This substance possesses a wide range of pharmacological properties and is used in medicinal plants for the treatment of Parkinson’s disease, rheumatoid arthritis, and diabetes. It slows the progression of mitochondrial diseases by stimulating the activity of anti-aging biomarkers (SIRT1 and SIRT6) and PGC-1β in the hypothalamus and may be a promising candidate for preventing diseases of aging. The molecular mechanisms include inhibition of the NF-κB signaling pathway, reduction of caspase activity (anti-apoptotic effect), activation of autophagy, and regulation of signaling pathways involved in cholesterol suppression [133] (Figure 6).

**Figure 7:**
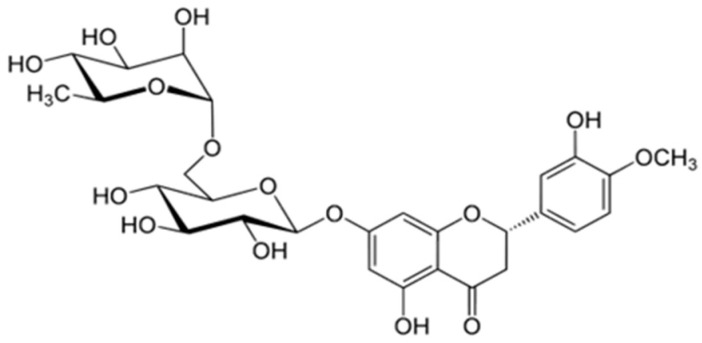
Structural formula of hesperidin.

Hesperidins are flavonoids obtained primarily from citrus peels. They act as antiglycating agents, which is important for the prevention of atherosclerosis. They also reduce the levels of pro-inflammatory interleukins and matrix metalloproteinases. They are a potent lymphotropic agent (Figure 7) [134].

**Figure 8:**
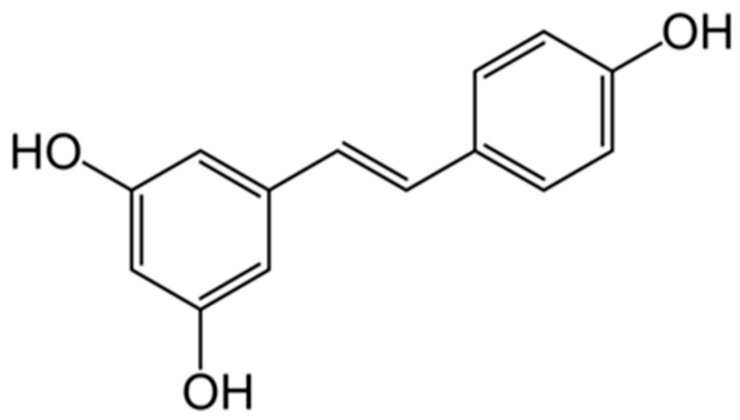
Structural formula of resveratrol.

Resveratrol is found in the skins of grapes and other fruits, cocoa, and nuts. It can extend lifespan in many animal models, primarily through the induction of autophagy, a reduction in oxidative stress, and neuroprotection. The key molecular mechanisms underlying these effects include activation of the longevity-associated protein SIRT1, stimulation of AMP-activated protein kinase (AMPK), enhancement of antioxidant defenses, suppression of pro-inflammatory signaling pathways (NF-κB), and modulation of estrogen receptors. Collectively, these processes protect cells from age-related damage, improve metabolic function, and exhibit anticancer potential (Figure 8) [135,136].

**Figure 9:**
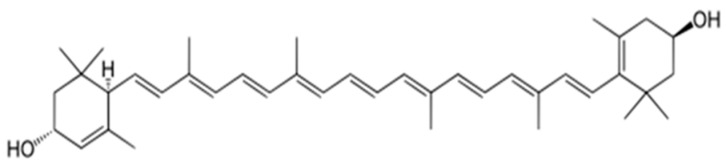
Structural formula of lutein.

Lutein (luteolin) is a pigment belonging to the xanthophylls—a group of oxygen-containing carotenoids—a plant compound with powerful antioxidant and anti-inflammatory properties. Lutein accumulates differently in different tissues: its highest concentration is observed in the eye, especially in the retina (10,000 times higher than in blood plasma), where it prevents oxidative damage to retinal cells. Luteolin also supports brain and cardiovascular health, and helps reduce uric acid levels. It is found in many fruits, vegetables, and medicinal plants, such as parsley, celery, broccoli, and chrysanthemum. Decreased catalase and superoxide dismutase activity regulates oxidative damage and lipid peroxidation. This is due to the process mediated by luteolin’s effect on NF-kB and mitogen-activated protein kinase (MAPK) activity (Figure 9) [137].

Lymphatic plants are a group of medicinal plants that help improve the functioning of the lymphatic system, including stimulating lymph flow and reducing interstitial congestion. These include burdock root, echinacea, horsetail, birch leaves and buds, St. John’s wort, and other species traditionally included in herbal teas used for inflammatory and congestive conditions. A common feature of these plants is the presence of phenolic compounds with anti-inflammatory and antioxidant activity.

Burdock root (*Arctium lappa* L.) is an effective anti-inflammatory agent. It helps mice with acute ear edema and rats with paw edema. The anti-inflammatory properties are attributed to the lignans lappaol F, diarctigenin, and arctigenin, which suppress nitric oxide synthesis in macrophages. Nitric oxide is known to be involved in inflammatory reactions in rheumatoid arthritis, autoimmune diseases, chronic inflammation, and atherosclerosis [138]. Burdock root is a component of herbal formulations traditionally employed to promote lymphatic drainage.

Of greatest practical interest are lignans with antitumor activity (podophyllotoxin and arctiin), matairesinol, 7-hydroxymatairesinol, diglucoside secoisolariciresinol, secoisolariciresinol, CNS-stimulating compounds (schisandra lignans), and hepatoprotectors (bicyclol and milk thistle lignans). Some lignans have estrogenic activity. Lignans are also antioxidants. Its mechanism of action is based on the inhibition of Akt and NF-B signaling pathways, regulation of nitric oxide synthesis, and reduction of the level of “stress” proteins, which blocks the proliferation of cancer cells and reduces inflammation [139].

A reduction in oxidative stress may contribute to maintaining the functional activity of lymphatic structures [140].

*Adonis vernalis* L. (*Spring Anemone*) is approved for medicinal use. The active ingredients are cardiotonic glycosides from the cardenolide group: derivatives of strophanthidin, adonitoxigenin, adonitoxol, and strophadogenin. The main ones are cymarin, K-strophanthin-β, adonitoxin, and K-strophanthoside. Adonis is included in a number of complex cardiac agents. Adonis preparations strengthen and slow heart contractions, increase stroke volume and cardiac output, and eliminate congestion in patients; they have a stronger calming effect on the central nervous system than other glycosides. Its stimulating effect on interstitial humoral transport and lymphatic drainage has been demonstrated. Thus, the molecular mechanism of action of *Adonis vernalis* L. is the modulation of cellular signaling pathways, including NF-kB and antioxidant mechanisms [141]. Origanum vulgare L. contains tannins and ascorbic acid; thymol—50 (according to other sources, up to 44 [14]); carvacrol; bi- and tricyclic sesquiterpenes—12.5; and geranyl acetate—2.6–5.

*Origanum vulgare* L. is used in pectoral, diaphoretic, and carminative infusions, and for colds and other respiratory ailments as an anti-inflammatory and expectorant. Origanum vulgare extract is included in the drug “Urolesan” [126]. It is known to stimulate lymphatic drainage and lymph flow. *Origanum vulgare* L. exhibits antioxidant, anti-inflammatory, and antimicrobial activities mediated by modulation of NF-κB and Nrf2 signaling pathways, as well as membrane-disruptive effects of its essential oil constituents, including carvacrol and thymol [141,142].

Almaty hawthorn (*Crataegus almaatensis Pojark.*) has been shown to have beneficial effects on cardiac weakness following acute illnesses [143]. It is widely used for cardiac disorders and to enhance urination. Many beneficial substances found in the leaves, berries, and other parts of hawthorn possess powerful antioxidant properties [144].

*Crataegus almaatensis* is a native Kazakh species traditionally used in cardiotonic folk medicine. Furthermore, chemical characterization of the plant leaves revealed that they contain flavonoid glycosides (quercitrin, hyperoside, and afzelin) specific to Crataegus species, which are involved in cardioprotection. Pharmacological studies have revealed vascular and anti-inflammatory effects of the extracts. Water fractions, in particular, enhanced vascular responses to norepinephrine and also exhibited vasodilatory effects, suggesting possible vascular involvement. Furthermore, the extract reduced inflammatory cell infiltration and the release of the pro-inflammatory cytokine IL-1β [145].

In our experiments, *Hawthorn* increased lymph flow by 50% [146]. Thus, the observed increase in lymph flow appears to result from enhanced drainage of the lymphatic system.

*Ziziphora Bungeana.* The Ziziphora genus is one of the most popular in this regard (aromatic species) within the Lamiaceae family, as it is present in various regions of Eurasia and North Africa. Several species of this genus are found in Kazakhstan; *Ziziphora bungeana* is known as a folk medicine. In addition to essential oils, plants of this genus contain phenolic acids, triterpenoids, and various flavonoid groups distinct from Lamiaceae. The antioxidant, antimicrobial, and immunomodulatory activities that have been theoretically tested may be related to the presence of these elements [147]. The plant has been indicated in traditional Kazakh medicine for the prevention of inflammatory and cardiac diseases, consistent with its proven biological properties [148]. *Zizifora bungeana* extracts are rich in antioxidants, reduce free radical production, and exhibit significant tyrosinase inhibition. Gram-positive bacterial strains were particularly sensitive to the antibacterial effect. At the same time, the extracts were non-toxic to normal cells and did not cause hemolysis, demonstrating biosafety. These effects indicate that *Zizifora bungeana* is a source of compounds that influence inflammation and immune defense processes, the mechanisms of which are also closely linked to the functioning of the lymphatic system [148].

Various articles examine the effects of *Zizifora bungeana* components on the cardiovascular system. Sources report cardiotonic and antiarrhythmic properties, and some plant metabolites influence microcirculation and oxidative stress. Essential oils include thymol, carvacrol, linalool, terpinen-4-ol, borneol, and 1,8-cineole—compounds believed to regulate vascular tone and endothelial function. Further evidence for its antioxidant and vascular protective properties is provided by its flavonoid profile (apigenin, chrysin, acacetin, and linarin) [149,150].

Biological compounds such as betulin, β-sitosterol, and the rarely isolated triterpene 3β-acetoxyolean-11-ene-28,13β-olide have been described as a source of compounds influencing inflammation, vascular reactivity, and lymphatic drainage [151]. In our experiments, *Ziziphora bungeane* increased lymph flow by 43% [146]. This increase in lymph flow can be explained by the complex effect of *Zizifora bungeana* compounds on the vascular and lymphatic system.

Cherry stalks (*Prunus cerasus* L.) accelerate metabolism and promote the gentle removal of excess fluid. However, the extract does not deplete potassium, unlike other diuretics. Cherry stalks enhance the circulation of interstitial fluid, stimulate the elimination of toxins and excess fluid, promote internal detoxification, and promote tissue drainage. A decoction with a strong diuretic effect, removing urea and urates, is used for edema, gout, dropsy, urolithiasis, uric acid diathesis, hypertension, and diarrhea. It has antiseptic and anti-inflammatory properties. Cherry stalks contain iodine, tannins, acids (citric and malic), dextrose, sucrose, quercetin, coumarins, and other active components. The stalks are filled with tannins, citric and malic acids, dextrose, quercetin, and fructose, which are easily digested by diabetics. Coumarins, present in the stalks, prevent blood clots. *Prunus cerasus* L. peduncles exhibit antioxidant, anti-inflammatory, and diuretic activities mediated through modulation of NF-κB and Nrf2 signaling pathways, as well as regulation of renal excretory function and uric acid metabolism [151].

*Hedysarum neglectum* contains flavonoids, catechins, saponins, tannins, xanthines, and alkaloids. *Hedysarum neglectum* has a wide range of pharmacological effects: anti-inflammatory, antitumor, immunostimulant, and tonic effects. Some representatives of this genus have also been found to have antiviral and antibacterial activity. *Hedysarum neglectum* exhibits antioxidant, anti-inflammatory, and immunomodulatory activities mediated through modulation of NF-κB and Nrf2 signaling pathways, as well as regulation of stress-response systems associated with the hypothalamic–pituitary–adrenal axis [152]. Our previous studies showed that Echinacea purpurea stimulates interstitial humoral transport and lymph flow by 50% [146].

*Echinacea purpurea* (L.) *Moench* contains micro- and macroelements, lipophilic substances, phenolic compounds, and polysaccharides. Echinacea purpurea is a key immunomodulator and adaptogen, which increases the body’s resistance to adverse environmental factors. The plant has antimicrobial activity, prevents inflammation in the lymph nodes, and improves lymph drainage from tissues [153].

Research by Aucoin and colleagues in 2021 confirmed that Echinacea purpurea extracts can modulate the production of cytokines, including IL 1β, IL 6, TNFα, and IFNγ, affecting macrophages, dendritic cells, and T lymphocytes [154]. This makes the plant an important candidate for study in the context of lymphatic system regulation.

*Stinging nettle* (*Urtica dioica* L.) leaves contain tannins; vitamins C, K, and B; the glycoside urticin; iron salts; carotenoids; pantothenic acid; protoporphyrin; sitosterol; scopoletin; histamine; chlorophyll; and phytoncides. These also have tonic effects, enhance basal metabolism, and improve muscle tone, which is essential for enhancing lymph flow. *Urtica dioica* L. exhibits antioxidant, anti-inflammatory, diuretic, and hypoglycemic activities mediated through modulation of NF-κB and Nrf2 signaling pathways, as well as regulation of glucose metabolism and renal excretory function [155].

*Bergenia crassifolia* (L.) Fritsch. Bergenia preparations have hemostatic, astringent, anti-inflammatory, hepatoprotective, and antimicrobial properties. They strengthen vascular walls, moderately lower blood pressure, and slightly increase heart rate. *Bergenia crassifolia* (L.) Fritsch exhibits antioxidant, anti-inflammatory, and antimicrobial activities mediated through modulation of NF-κB and Nrf2 signaling pathways, as well as tannin-dependent astringent effects on gastrointestinal mucosa [150]. In our experiments, bergenia increased lymph flow by 43% [146].

Lady’s Mantle (*Alchemilla vulgaris*). It is known that the aerial parts contain tannins (7.2–11.3%) and catechins. In the green parts of the plant, the tannin content ranges from 7.5 to 9.4%; flavonoids, phenolic acids and their derivatives (luteic and ellagic acids), lignin, lipids, and coumarins are also present. Medicinal preparations from lady’s mantle exhibit anti-inflammatory, astringent, expectorant, wound-healing, diuretic, and lactogenic effects. Regular oral intake of leaf infusions leads to a reduction in blood cholesterol levels. In gynecology, lady’s mantle is used as a hemostatic, helps normalize the menstrual cycle, and supports the treatment of female reproductive disorders. Its pronounced lymphotropic activity has also been documented [13]. *Alchemilla vulgaris* exerts anti-inflammatory and antioxidant effects through inhibition of NF-κB signaling, activation of the Nrf2/ARE pathway, reduction of ROS levels, and modulation of COX/LOX pathways, contributing to its cytoprotective and astringent properties.

Great Burnet (*Sanguisorba officinalis*). In scientific medicine, the rhizome and root of great burnet are used as medicinal raw materials. The rhizomes contain tannins (up to 23%), essential oil, and saponins. The roots contain 16–17% tannins, while callus may contain up to 23% of pyrogallol-type tannins; the leaves contain ascorbic acid (up to 0.92%). Infusions and decoctions of great burnet exhibit bactericidal, astringent, and strong hemostatic effects. Studies by Levin Yu.M. have shown its significant lymphotropic activity [13]. *Sanguisorba officinalis* L. exerts its anti-inflammatory, antioxidant, and cytoprotective effects through inhibition of NF-κB signaling, activation of the Nrf2/ARE pathway, modulation of MAPK cascades, regulation of apoptosis, and reduction of oxidative stress.

Hill saltwort (*Salsola collina Pall.*) is the basis for the drug salsocollin, which has choleretic, anti-inflammatory, antioxidant, and immunostimulatory effects. It promotes liver cell regeneration (hepatoprotective action), strengthens blood vessels and capillaries, helps normalize blood pressure, has immunostimulatory and antioxidant properties, and slows the aging process. *Salsola collina Pall.* exhibits antioxidant, anti-inflammatory, hypoglycemic, and hepatoprotective activities mediated through modulation of NF-κB and Nrf2 signaling pathways, as well as regulation of glucose metabolism and oxidative stress responses [156,157].

*Rhubarb* (*Rhéum*) has long been eaten and used medicinally. Recent research shows that members of the *Rheum genus*, particularly *Rheum rhaponticum* and *Rheum rhabarbarum*, possess a wide range of biologically active compounds and significant therapeutic potential. Numerous phytochemical groups have been identified in their composition—stibenes, anthraquinones, flavonoids, and phenolic acids—which determine their antioxidant, anti-inflammatory, antimicrobial, and cardioprotective properties [158]. *Rheum L.* species exhibit antioxidant, anti-inflammatory, laxative, and hepatoprotective activities mediated through modulation of NF-κB and Nrf2 signaling pathways, as well as anthraquinone-dependent regulation of intestinal motility and water-electrolyte balance. Particular attention is given to the species *Rheum compactum* (syn. *R. altaicum*) and *R. tataricum,* included in the State Register of Medicines of the Republic of Kazakhstan. These species are characterized by high contents of tannins, anthraquinones, flavonoids, stilbenes, catechins, and organic acids, which explains their broad therapeutic activity. Most species of the *Rheum genus* have the potential to produce domestic herbal remedies with anti-inflammatory, astringent, hemostatic, and antitumor effects [159].

According to Liudvytska et al. (2023), extracts of *R. rhaponticum* and *R. rhabarbarum* exhibit complex effects on the human hemostasis system. The study results demonstrated the anticoagulant effect of the plant extracts, expressed through the inhibition of key serine proteases of the coagulation cascade (thrombin and factor Xa), as well as a reduction in tissue factor-induced plasma coagulation. These data confirm the potential of Rheum species as sources of compounds capable of modulating vascular processes, which is particularly important in the context of age-associated disorders [160].

The herbal preparation KATREL (lingonberry, bergenia, currant, and rosehip leaves) has proven to be an effective lymphotropic agent, accelerating tissue fluid transport by more than 20 times [13].

However, despite expanding knowledge about the effects of drugs on tissue lymphatic drainage, information on the effects of drugs with antioxidant activity on lymphatic flow remains sporadic, and the mechanisms of lymphogonic activity are poorly understood [161].

Our studies examined the effect of a phytocomposition of Kazakh plants—*Ziziphora bungeane* (ZB), *Crataegus almatinica* (BA), *Echinacea* (E), *Hedysarum kopeckii* (K), and *Bergenia crassifolia* (BT)—on age-related changes in the composition of biological fluids (blood, lymph, and interstitial fluid) in old (22–24 months) white laboratory Sprague-Dawley rats. Administration of the phytocomposition for 3 months resulted in an 8% increase in interstitial fluid (IF) and plasma volume, as well as a 31% increase in lymph flow and a 41% increase in diuresis. Lipid levels in the blood and lymph decreased by 12%. The Clotting time increased, the viscosity decreased, and the lipid levels in the blood and lymph decreased. Increased levels of erythrocytes, leukocytes, immunoglobulins (except IgG), and lymphocyte subpopulations were also noted, which contributed to enhanced immune properties of the blood and lymph. Increased tissue hydration, the antiatherogenic properties of lymph and blood, and enhanced immune properties lead to the restoration of the drainage and detoxification function of the lymphatic system. Thanks to bioflavonoids, trace elements, and vitamins, the phytocomposition has a lymphotropic effect, changing the composition of blood, lymph, and interstitial fluid; stimulating fluid movement from the interstitium into the vascular bed; enhancing natural lymph detoxification; and enhancing the immune properties of the blood and lymph [82]. All the studied antioxidant drugs enhance interstitial humoral transport and the lymphatic drainage of tissues; however, the magnitude of their lymphostimulating activity in most cases does not correspond to their antioxidant potency. Mexidol and emoxypine significantly shorten the time taken for lymphotropic dye to be eliminated from the mesentery of mice [162,163].

Mexidol (ethyl methylhydroxypyridine succinate—a modified vitamin B6 molecule) is considered a universal antioxidant pharmacotherapy agent because it affects various components of oxidative stress: it inhibits the free radical oxidation of biomembranous lipids; actively reacts with lipid peroxide radicals, and primary and hydroxyl radicals of peptides; reduces NO levels; and increases the activity of SOD and other antioxidant enzymes [164]. However, Mexidol is not used in medical practice in Europe and America, as scientific evidence of its effectiveness is lacking.

However, we are interested in the lymphogonic effect of this drug. Thus, the use of Mexidol in combination with Chofitol in modeling exogenous intoxication in both mature and old mice leads to an increase in the rate of interstitial humoral transport and lymphatic drainage of tissues, a decrease in the concentration of malondialdehyde in the blood serum, and a decrease in the hematological index of intoxication, but does not bring these indicators to the levels recorded in intact animals of corresponding age. The effectiveness of therapy in restoring lymph flow and suppressing lipid peroxidation processes is more significant in mature mice, and its effectiveness in normalizing the hematological index of intoxication is greater in old animals [165,166].

## 11. Conclusions

The mechanisms of aging are determined by the following key features: genomic instability, telomere attrition, epigenetic changes, impaired proteostasis, impaired macroautophagy, impaired nutrient uptake, mitochondrial dysfunction, cellular senescence, stem cell depletion, impaired intercellular communication, chronic inflammation, dysbiosis, hormonal dysfunction, and impaired interstitial humoral transport and lymph flow. At the tissue and organ level, changes in interstitial humoral transport and lymph flow are very important, as tissue congestion leads to the accumulation of toxic metabolites, which affects the body at the cellular and subcellular levels. Herbal remedies, due to their flavonoids, phenolic compounds, terpenoids, glycosides, polysaccharides, and other compounds, inhibit the free radical oxidation of biomembrane lipids, reduce NO levels, increase antioxidant enzyme activity, eliminate inflammation, and normalize interstitial humoral transport and lymph flow. The lymphatic system and its components, including interstitial fluid and lymph, constitute the internal environments of mammals and humans. It is within this medium that intercellular processes, such as membrane contacts, occur, and regulatory factors as well as other cellular metabolites are secreted into it. As a liquid milieu within the cellular microenvironment, it is homeostatically regulated by the organism. This provides a rationale for its direct or indirect involvement in aging mechanisms of various types.

Herbal remedies containing burdock root, cherry stalks, echinacea, adonis, oregano, ziziphora, crataegus, Katrel, and Mexidol, along with antioxidant activity, enhance interstitial humoral transport and lymphatic drainage. This can justify the positive effects of these drugs on the processes occurring in the body during aging. The role of interstitial humoral transport and lymph flow, along with renal mechanisms, is the basis of the body’s fluid homeostasis. The use of drugs and herbal remedies with lymphatic, anti-inflammatory, antioxidant, and cytoprotective properties can ensure normal physiological functioning, reduce tissue congestion, and improve quality of life in old age.

It is recommend to regulate interstitial humoral transport and lymph flow through the qualitative and quantitative selection of medicinal plants, taking into account their influence on lymphatic system function.

### Recommendations for Future Research

Promising areas for further research include studying the role of interstitial humoral transport and lymph flow in the key mechanisms of aging, as well as their relationship with chronic inflammation, oxidative stress, and cellular senescence. Research is needed on the molecular mechanisms by which herbal remedies act on the lymphatic system, including their effects on antioxidant and anti-inflammatory signaling pathways.

Experimental and clinical studies aimed at assessing the effectiveness of herbal remedies in restoring tissue drainage and water homeostasis, and improving quality of life in old age, taking into account the personal selection of medicinal plants, remain relevant.

## Data Availability

The data presented in this study are available from the corresponding author on request.
